# Supplementary material for: MetaRibo-Seq measures translation in microbiomes
Source: Nat Commun. 2020 Jun 29;11:3268. doi: 10.1038/s41467-020-17081-z (PMC7324362; doi:10.1038/s41467-020-17081-z)
Supplement: Supplementary file 10 — Supplementary Data 7 [file 41467_2020_17081_MOESM10_ESM.zip › File2/Confidence_VeryHigh_Taxonomy/36775_out.krona.html]

Javascript must be enabled to view this page.

members
magnitude
magnitudeUnassigned
count
unassigned
taxon
rank

36775\_out

5

2
superkingdom
5

1239
phylum
5

5
class
186801

5
order
186802

31979
family
5

1649459
genus
5


SRS017521\_contig\_number\_contig-100\_604.100667SRS019601\_contig\_number\_5601SRS144362\_contig\_number\_contig-100\_983.233252SRS146888\_contig\_number\_14444SRS148196\_contig\_number\_contig-100\_1418.113843
species
154046
5
